# Recent Progress, Challenges, and Trends in Polymer-Based Sensors: A Review

**DOI:** 10.3390/polym14112164

**Published:** 2022-05-26

**Authors:** Mir Waqas Alam, Shahidul Islam Bhat, Hassan S. Al Qahtani, Muhammad Aamir, Muhammad Nasir Amin, Mohd Farhan, Sara Aldabal, Muhammad Shuaib Khan, Ishtiaq Jeelani, Allah Nawaz, Basma Souayeh

**Affiliations:** 1 Department of Physics, College of Science, King Faisal University, Al-Ahsa 31982, Saudi Arabia; saldabal@kfu.edu.sa (S.A.); bsouayeh@kfu.edu.sa (B.S.); 2 Corrosion Research Laboratory, Department of Applied Chemistry, Aligarh Muslim University, Aligarh 202002, India; shahid.bhat19@gmail.com; 3 EXPEC Advanced Research Centre, Saudi Aramco, Dhahran 31311, Saudi Arabia; hassan.alqahtani.2@aramco.com; 4 Department of Basic Sciences, Preparatory Year Deanship, King Faisal University, Al-Ahsa 31982, Saudi Arabia; msadiq@kfu.edu.sa (M.A.); mfarhan@kfu.edu.sa (M.F.); 5 Department of Civil and Environmental Engineering, College of Engineering, King Faisal University, Al-Ahsa 31982, Saudi Arabia; mgadir@kfu.edu.sa; 6 International Research Center for Renewable Energy (IRCRE), State Key Laboratory of Multiphase Flow in Power Engineering (MPFE), Xi’an Jiaotong University, 28 West Xianning Road, Xi’an 710049, China; m.shuaibkhan@mail.xjtu.edu.cn; 7 Department of Medicine, University of California, 9500 Gilman Drive, La Jolla, San Diego, CA 92093, USA; ishtiaqjeelani66@gmail.com; 8 Department of Molecular and Medical Pharmacology, Faculty of Medicine, University of Toyama, Toyama 930-0194, Japan; nawaz@med.u-toyama.ac.jp

**Keywords:** shape memory, polymer-based sensors

## Abstract

Polymers are long-chain, highly molecular weight molecules containing large numbers of repeating units within their backbone derived from the product of polymerization of monomeric units. The materials exhibit unique properties based on the types of bonds that exist within their structures. Among these, some behave as rubbers because of their excellent bending ability, lightweight nature, and shape memory. Moreover, their tunable chemical, structural, and electrical properties make them promising candidates for their use as sensing materials. Polymer-based sensors are highly utilized in the current scenario in the public health sector and environment control due to their rapid detection, small size, high sensitivity, and suitability in atmospheric conditions. Therefore, the aim of this review article is to highlight the current progress in polymer-based sensors. More importantly, this review provides general trends and challenges in sensor technology based on polymer materials.

## 1. Introduction

Smart materials are substances that respond to given impulses from external sources via physical or chemical stimuli, resulting in changes in material characteristics. In the current scenario, considerable interest is being devoted to polymers due to their reversible or irreversible nature (changing properties under external stimuli), such as pH, light radiation, temperature, and electric/magnetic fields [1]. Smart polymers exist in the form of solutions, gels, self-assembled nanoparticles, films, or solids [2,3,4,5]. Researchers are utilizing already known characteristics of these materials in more complex issues, such as the controlled delivery of drugs and genes, catalysis, detection and imaging, adaptive coatings, or self-healing materials [6,7,8]. Polymer-derived materials can be easily processed and biocompatible [9,10,11,12]. Polymer-based sensors have gained considerable interest, which is evident from the number of published research articles and the amount invested in this branch of study [13]. Sensors derive information about the environment in which they are employed (locally/remotely). Polymer-based materials can be tuned through appropriate synthetic or modification techniques, making polymers prominent in sensing devices [14] with diverse applications [15].

Generally, analytical techniques for a sensor require sophisticated instrumentation facilities, trained manpower, and scientific protocols that pose severe limitations to use by common people. Hence, the topic of greater importance is the development of cheap sensors employing a user-friendly approach. The most promising technologies for sensor and biosensor processing are polymer-derived technologies. The common polymeric materials used in sensing devices are hydrogels, conducting polymers, molecular imprinting polymers, and composites and nanocomposites [16,17,18,19]. The materials employed in these polymer sensors enhance the target molecular recognition as basic support for immobilization of various functionalities (metal nanofillers, dyes, etc.,) and by altering the physical/chemical properties, hence allowing target analyte detection [20,21]. Another polymer-based sensor advantage is the chance of modifying their chemical characteristics in such a way that their degradation resistance, flexibility, reactivity, and biodegradability are tuned [22].

Thus, in light of the above-mentioned facts, the current review discusses various types of polymer matrices for different sensing materials and the applications of various polymer sensors. Moreover, much attention has been focused on the challenges and trends involved in the current field of study. Examples of various types of polymer-based sensors (natural and synthetic) have been discussed in this review.

## 2. Biopolymer-Derived Sensors

**Figure 1:**
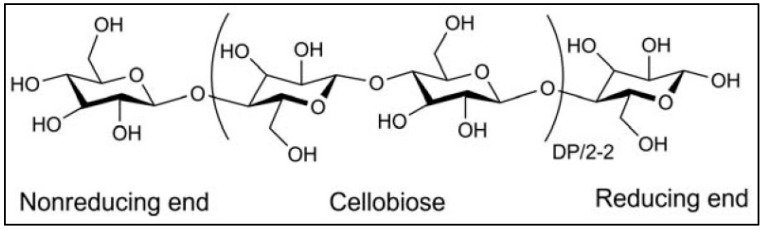
Structure of cellulose reprinted with permission from ref. [24]. Copyright 2010 American Chemical Society.

Natural (macromolecular) polymers are generated from plants/animals that are used in a variety of applications, including the cosmetic industry, pharmaceutics, etc. They are inexpensive, adjustable, widely accessible, degradable, and biocompatible. Nevertheless, microbes can destroy them, the degree of hydration varies, and metal ions can contaminate the polymer surface [23]. Plant-derived materials include starch, cellulose, acacia gum, hemicellulose, inulin, glucomannan, and pectin, whereas animal-derived materials include chitin, chitosan, and alginate [23]. This homopolymer is made up of D-anhydroglucopyranose units connected by a (1–4)-glucosidic linkage (Figure 1) [24].

Cellulose is colorless, odorless, adsorbent, and hydrophilic due to the paper’s vivid, high-contrast, colorless backdrop, which is suitable for well-appreciated color changes, and the surface groups get functionalized easily to generate orimetric sensors. Alberti et al. [25] designed a Fe(III)/V(IV)-based sensor from cellulose functionalized by a powerful chelating agent (deferoxamine [DFO]) that generates stable and colorful complexes. The DFO paper sensor was created using a method proposed earlier by Takagi et al. [26], which comprises hydroxyl group halogenation and later reaction with molecules of deferoxamine. Following Fe(II) exposure, the RGB properties of the DFO–papers pictures were changed. Disposable sensors could be generated from nanocellulose generated from nanofibers, nanostructured materials, or microorganisms. Cellulose nanofibers (CNFs) can be obtained and purified from cellulose fibers of lignocellulosic materials (for example, wood, and agricultural residue) using a variety of mechanical and chemical technologies [27]. Chauhan and colleagues [28] developed a new optical pH sensor based on dye-functionalized nanocellulose production in a single step. The resultant nanomaterial is covalently linked with a Remazol dye. The dye created a stable suspension where the color changed from bright red to purple as the pH was altered from acid to alkaline. A disposable stick was made using adhesive tape to adhere a section of the lignocellulose film to a plastic strip. It can detect pH changes in a reversible and quick manner. Chitosan is another common biopolymer. This macromolecule is a polysaccharide derived from the deacetylation of chitin, a molecule found in the exoskeleton of shellfish [29]. It exhibits outstanding adhesive properties, film permeability, water permeability, dimensional stability, and biocompatibility. Chitosan, having a pKa of 6.3, is deployed as an electron acceptor in surface-modified sensors for anions due to amines on the polymer’s surface. It is a fantastic conducting polymer that has attracted significant attention as a sensor and is made from modified seafood waste such as crab and shrimp. It is a special substance that can be used in microdevices because of its high density of amine groups, which provide active bonding sites [30]. Chitosan has great properties for use as an electrochemical and gas sensor. Furthermore, it has been employed as a biosensor because of its remarkable film-forming characteristics and its ability to maintain its inherent features. Chandrasa Karan et al. [31] used an electrochemical deposition approach to successfully create a chitosan-based ammonia sensor that has all the necessary characteristics of a dependable sensor, such as repeatability, sensitivity, recovery, and speed in response to ammonia exposure. Furthermore, a sensor’s capacity to function at room temperature with minimal energy consumption and fabrication costs makes it a dependable sensor that can be employed in a variety of applications.

A quick self-healing bio-based polymer was utilized to examine perspiration in humans in real time, with the data being successfully sent to a smartphone. Perspiration detector threads can be knitted into fabric or connected with a wireless flexible printed circuit board (FPCB) for real-time sweat monitoring. The self-healing potentiometric ion-sensing thread is made by covering carbon fiber thread (CFT) electrodes with a strong citric acid-based supramolecular polymer capable of extraordinarily fast self-healing within 30 s at ambient temperature. These flexible threads may simultaneously detect electrolytic potassium and sodium ions. After 30 s of healing, the sweat sensors demonstrate exceptional sensitivity and impressive self-healing efficiency (>97.0%). A wireless electrical circuit board containing the built wearable ion sensor system was checked on humans while they operated a bicycle [32].

Gogoi et al. [33] produced and characterized bio-based polymers derived from curcumin, polycurcumin acrylate (PCUA), and polycurcumin methacrylate (PCUMA). Picric acid and nitrobenzene are chemically sensitive to these polymers. PCUMA is more effective than PCUA as a nitroaromatic vapor sensor. At ambient temperature, the polymer is also a good nitroaromatic compound detector. The presence of aromatic rings in the monomer allows for graft polymerization and copolymerization, which could lead to a novel nitroaromatic sensor with an improved response. This sensor has the advantage of being able to detect a variety of nitroaromatic chemicals while also being reversible.

## 3. Synthetic Polymer-Based Sensors

### 3.1. Polymers Using Molecular Imprinting (MIPs)

The template-induced synthesis of chemically synthesized transmitters in a polymer is known as molecular imprinting. Specific recognition sites generated by molecular imprinting offer remarkable properties, such as good specificity durability as well as competitive prices, providing compounds tempting substitutes to natural transmitters. Advances in nanotechnology and polymer science have also helped in the performance of screen-printed carbon polymer (MIP) sensing devices. The importance of molecular recognition in biological processes cannot be overstated. It is currently the subject of several studies due to its catalysis and sensing applications. Modern sensor studies aim at developing synthetic receptors that could provide natural interactions between antibodies and antigens, taking both sensitivity and specificity into consideration. The electrochemical deposition process includes creating recognition sites for polymer molecules, which function as templates.

**Figure 2:**
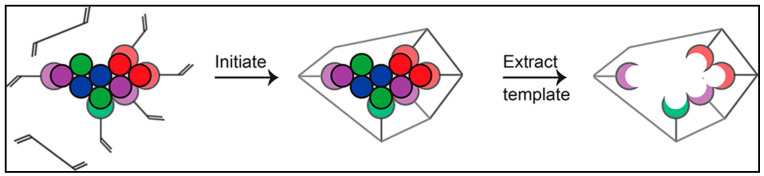
General process of imprinting. Reprinted with permission from reference [30]. Copyright 2017 American Chemical Society.

The linkages between the framework and functional monomers were also maintained throughout polymerization and were stabilized by crosslinking in the polymer [34]. A general process of imprinting is shown in Figure 2.

**Figure 3:**
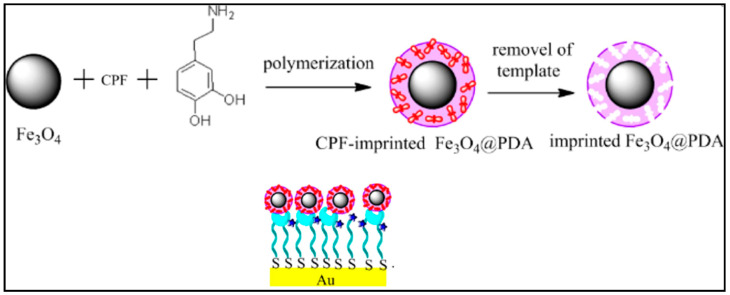
(**Top**) Schematic imprinted magnetic nanoparticle synthesis. (**Bottom**) Representation of imprinted nanoparticles attached to thiol ligands on a gold surface for sensing. Reprinted with permission from the reference [36]. Copyright 2019 American Chemical Society.

The interaction of the monomer with the template tends to form a cavity around the molecular template; the template is later removed, thus leaving behind the imprinted cavity for target molecular rebinding. The sensor sensitivity has a value of 2.1 W/% RH and an 89 percent repeatability with recovery and response times 155 and 25 s correspondingly. As more than just an outcome, the developed MIP may detect the target analyte preferentially in the template-derived locations [35,36]. A molecularly imprinted polymer magnetic nanoparticle system is depicted in Figure 3.

Enzymes, proteins, bacteria, viruses, metal ions, poisons, and other templates may all be imprinted with molecular imprinting polymers. They are promising materials, particularly in sensor systems, due to their precise recognition sites.

Multi-threading sensors seem to be effective and inexpensive biomolecule sensors. They can be employed in electrical, chemical, optical, and electromechanical monitoring modes [37]. The interaction of an electrolyte and receptors on the surface of an electrode is the basis for electrochemical sensors. In one intriguing study [38], for example, a voltametric theophylline-based sensor for sol-gel immobilized (MIPs) is given. Theophylline has been used to treat infections and serious lung sickness for over 70 years due to its low cost and broad availability. Microspherical and macroporous MIP particles were immobilized on the surface of a carbon electrode using the sol-gel process, together with graphite (the conducting medium). An alternating heartbeat-modified electrode is utilized as a voltametric method to quantify methylphenidate at concentrations as low as 1 M, yielding findings that are comparable or lower than those reported by electrochemical methods. Another unusual yet intriguing work [39] created an MIP-based duplex novel nucleotide recognition sensor. In one case, a cocaine potentiometric MIP nanoparticle-based sensor was developed [40]. Cocaine is a popular recreational drug; its excessive usage causes symptoms such as internal bleeding, depression, and respiratory arrest, and its use has economic and social ramifications. As a result, it is necessary to develop sensitive and easy cocaine detection technology, especially for either medical or investigative applications. This novel potentiometric biosensor has been built on functionalized polymer nanoparticles (nano MIPs), which are created by a decent optimization phase. A novel potentiometric sensor is built on nanocomposite membrane polymer nanoparticles (nano MIPs) which are created by a solid-phase imprinting process. Nano MIPs manufactured using functional monomer as the acrylamide exhibited the maximum yield and responsiveness to cocaine which is the reason for their choice in device fabrication. A recent study [41] presented temperature-sensitive electrical and chemical sensors derived from MIPs for serum albumin detection in bovines. A hydrogel (thermoresponsive)-derived biosensor is based on a layer formed on the surface of a glassy carbon electrode by free radical polymerization. When external temperature stimulation is administered, a permanent structural change in MIP promotes bovine serum albumin (BSA) sensing. The BSA sensor performed admirably in terms of selectivity, stability, recovery, and repeatability.

MIP-based optical sensors are classified into two types: (i) MIP affinity sensors and (ii) MIP optoelectronic sensors. For MIP affinity sensors, the devices are capable of detecting analytes possessing properties such as fluorescence, absorbance, and refractive index. For optoelectronic sensors, binding at the analyte/MIP site leads to increased absorbance at a certain wavelength, fluorescence quenching, or a change in refractive index [42,43]. Wren and colleagues [44,45] created a fluorescent optical fiber chemical sensor for cocaine. A fluorescent probe for optical fibers was made by synthesizing a rationally selected fluorophore and then incorporating it into a molecularly imprinted polymer. The amount of fluorescence emitted is proportional to the amount of cocaine present in a sample. This sensor is promising with a detection limit of about 1 M, and it takes research in a new direction by providing a rapid and low-cost approach that could be beneficial in drug forensics. Furthermore, they talked about certain MIP-based electrical, chemical, and optical sensing devices, emphasizing their applicability to real-world samples. MIPs moved beyond simple technology demonstrators. The goal at hand should be to scale up production, increase robustness, and find a sustainable market.

### 3.2. Sensors Based on Conducting Polymers

**Figure 4:**
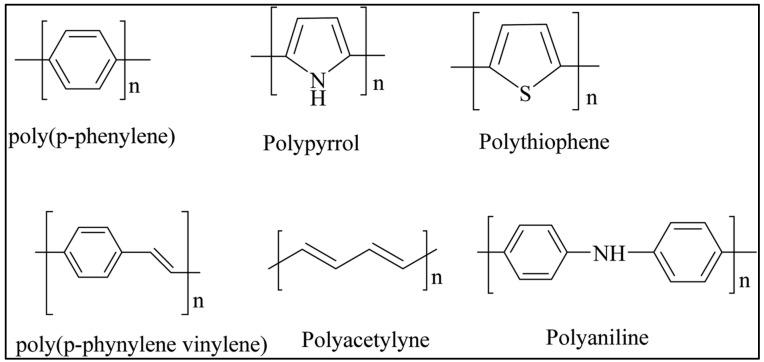
Most utilized conducting polymers. Reprinted with permission reference [49]. Copyright 2021 Royal Society of Chemistry.

Compounds have been differentiated via their potential to delocalize their pi electrons. Polyacetylene could be turned into a conducting polymer upon doping with Br_2_ and I_2_ during the first time by MacDiarmid, Shirakawa, and Heeger in 1972 [46,47]. In 2000, this invention was recognized with the Nobel Prize in Chemistry. Conducting polymers provide numerous advantages, including the ability to be doped to display metallic and semiconducting properties. The materials can combine plastic and electrical properties. They may be altered, decomposed in non-polar solvents, and printed using low-cost techniques. However, there are certain disadvantages, such as a lack of long-term stability. They have a wide range of applications due to their characteristics, including supercapacitors, nanocoatings, catalysis, biomedicine, and sensors [48,49]. Polyaniline, polyacetylene (PA), polypyrrole, poly(para-phenylene), and polyfuran were the most often utilized conducting polymers (Figure 4).

Due to their redox activity, this class of polymers is used as a gas detector in sensing. Performance-enhancing drugs can be a p-type or n-type configuration and can change the polymer from a semiconductor to a conducting one. In p-type polymers, the electron migration is from the HOMO-LUMO polymer (chain HOMO) to doping agent LUMO. This transition leads to the creation of polymer holes, causing them to lose electrons. When an oxidized polymer (p-type) is exposed to a reducing agent (CO, NH_3_, CH_4_, H_2_, H_2_S, acetone, or ethanol), its resistivity rises; conversely, when the polymer’s surface reacts with an oxidizing agent (NOx, CO_2_, SO_2_, O_2_, or O_3_), its resistivity reduces [50,51]. Korent et al. [52,53] reported an inexpensive NH_3_ sensor based on the polyaniline functionalization of a screen-printed electrode (SPE). The SPE was altered in this study by electrochemically polymerizing polyaniline in HCl. The compiled signal is produced by the reaction of the oxidized polymer substrate (PANIH^+^) and NH_3_; this electron exchange produces an electric flow.

PANIH^+^ + NH_3_ PANI + NH_4_^+^

Sensors built of conducting polymers are more accurate than GC/MS. Sensor device assembly should be mechanized, for example, using 3D technology, with a reaction time of a few seconds [54,55]. Forzani et al. [56,57] investigated a glucose oxidase-based electrochemical sensor (GOx). The sensing array is composed of junctions of nano polyaniline, where electropolymerization of monomer takes place in the presence of poly (acrylic acid). GOx increases glucose oxidation to gluconolactone, followed by enzyme degradation to GOx (FADH_2_). The reduced enzyme combines with the oxygen in the solution, resulting in the formation of GOxFAD and H_2_O_2_. The conductive polymer is oxidized by peroxide in the last phase.

glucose + GOx(FAD) → gluconolactone + GOx(FADH_2_)

GOx(FADH_2_) + O_2_ → GOx(FAD) + H_2_O_2_

PANIred + H_2_O_2_ → H_2_O + PANIox

**Figure 5:**
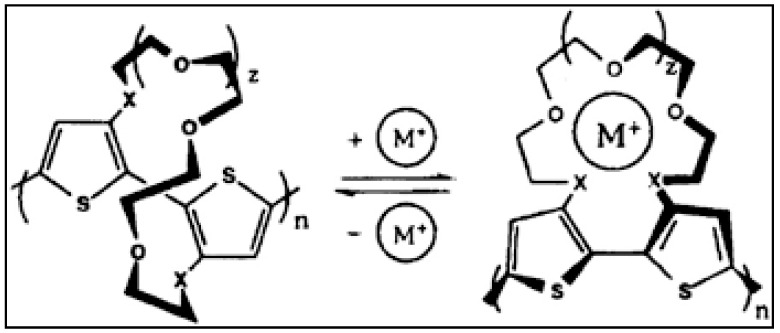
Crown ether modified polythiophene for sensing the metal cations of alkali. Reprinted with permission from the reference [58]. Copyright 1993 American Chemical Society.

Swager et al. [58] assembled a Na^+^-based sensor by conducting polymer polythiophene. The first part was carried out by modifying polythiophene with a crown ether. The polymer in an unbound state maintains molecular planarity by distortion of the pi electron cloud. On complex formation with Na^+^, there is a twist in the structure because a structure distortion takes place, leading to a decrease in conjugation and conductivity (Figure 5).

The glucose oxidase is then bonded to the polymeric surface. GOx promotes enzyme degradation to GOx(FADH_2_) and oxidation of glucose to gluconolactone. The reaction of the reduced enzyme and oxygen in the solution phase produces GOxFAD and H_2_O_2_. In the last step, the conductive polymer is oxidized by peroxide [59,60]. The conductivity of the polymer reduces when it is electrochemically oxidized in the presence of Na^+^. The impact is likeliest due to the higher inductive action of macrocyclic oxygens [61,62]. To summarize, conducting polymers are widely employed in all types of sensors due to their adjustable electronic properties, simplicity of polymerization, and inexpensive production costs.

### 3.3. Sensors Based on Acrylic Polymers

**Figure 6:**
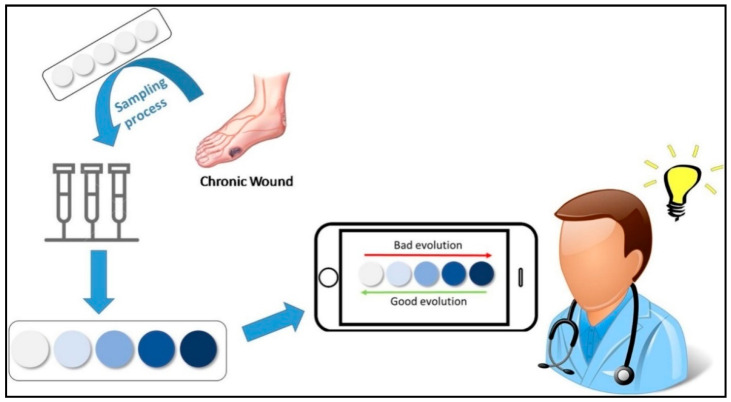
Monitoring chronic human wounds. Reprinted with permission from the reference [63]. Copyright 2021 Elsevier.

The use of acrylic polymers in sensing is attributed to the sensing functionalities that are covalently bound to the polymeric backbone, which can be tailored to process materials with diverse properties. The commonly used acrylic polymers employed for this purpose are derivatives of acrylamide, their copolymers, and meth/acrylic acid. Various functional groups can be incorporated into acrylic moieties for designing sensor-based materials [63]. Garcia et al. reported acrylic-based sensors with fluorescent/colorimetric properties for various analytes [64,65]. The strategy consisted of thin film polymer preparation by radical polymerization, where 1-vinyl-2-pyrrolidone served as the hydrophilic base, methyl methacrylate (MMA) monomer as the hydrophobic part, and a sensory unit (side group reactive moiety). In one of the works on polymers based on ninhydrin, the evolution of “chronic human wounds” was pictorially represented (Figure 6) [66].

The package includes a colorimetric polymer film that changes color when exposed to amino acids. The kit allows for the quantification of total amino acid content by examining the color properties of a sensory film (RGB) acquired from smartphone images. This gadget can aid in the diagnosis of chronic human ailments by providing an analytical procedure that is not influenced by subjective assessment [67,68]. It can create a pH polyacrylate-based sensor where polyacrylic acid (PAA) is obtained utilizing a free radical polymerization approach in aqueous media. The use of ultrasonic energy allows for more robust and environmentally friendly polymerization. PAA is then utilized as a capping agent in the synthesis of AgNPs in the absence of any UV/gamma radiations or any additional reducing agents. Another noteworthy work discussed the creation of an optoelectronic humidity sensor based on Sc(III) polyacrylics [69,70]. To investigate the sorption/desorption of humidity at normal temperature, nanostructured scandium polyacrylate was placed on flat borosilicate substrates. The use of ultrasonic energy allows for more robust and environmentally friendly polymerization. PAA is then utilized as a capping agent in the synthesis of AgNPs in the absence of any additional reducing agents or UV/gamma radiations. The resulting Ag-PA sol was used to detect pH with the naked eye. This study is an example of a feasible low-cost pH sensor based on a colorimetric smart polymer. Another noteworthy work discussed the creation of an optoelectronic humidity sensor based on Sc(III) polyacrylic [71]. To investigate the sorption/desorption of humidity at normal temperatures, nanoscandium-polyacrylate was placed on flat borosilicate surfaces. The sensor has a sensitivity value of 2.1 W/% RH and 89% repeatability; the sensor’s response and recovery times are 25 and 155 s, respectively. It appears to be a viable humidity-measuring gadget.

### 3.4. EVOH Polymers

**Figure 7:**
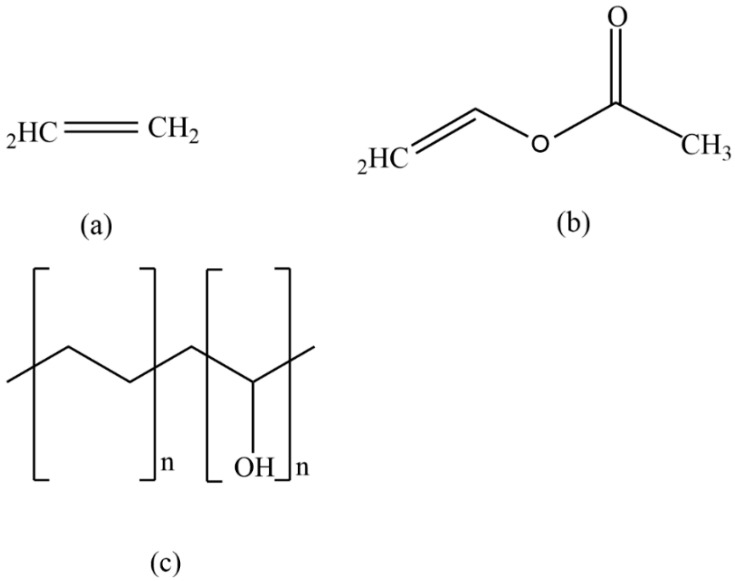
The structure of ethylene monomer (**a**), vinyl acetate monomer (**b**) and vinyl alcohol monomers (**c**)**.**

Because of its barrier qualities against gases and humidity, EVOH is a diblock copolymer consisting of ethylene and vinyl alcohol, which is extensively used in the food packaging and pharmaceutical sectors. When ethylene and vinyl alcohol monomers are copolymerized, the keto–enolic tautomeric balance moves to the aldehydic form (Figure 7), due to which the structure cannot be achieved. As a result, the most often employed synthetic process calls for ethylene and vinyl acetate as monomers, followed by hydrolysis [72].

EVOH is offered on a large scale in various ethylene proportions, viz., 27%, 32%, 38%, and 44%; the variable percentages of monomers result in divergent railing qualities in opposition to gases and solubility in organic solvents. Excess ethylene rates in the formation render the diblock copolymer very hygroscopic and, as a result, with less restraining characteristics when creating hydrogels [40]. It is critical to emphasize that the tacticity and novel functionalities on the polymer’s surface influence its solubility. EVOH is a great contender for sensor development due to its outstanding mechanical characteristics and the simplicity of surface functionalization. Cui et al. [41] used EVOH as a polymeric solid phase to construct a sensor for measuring Cu^2+^ in an aqueous solution.

**Figure 8:**
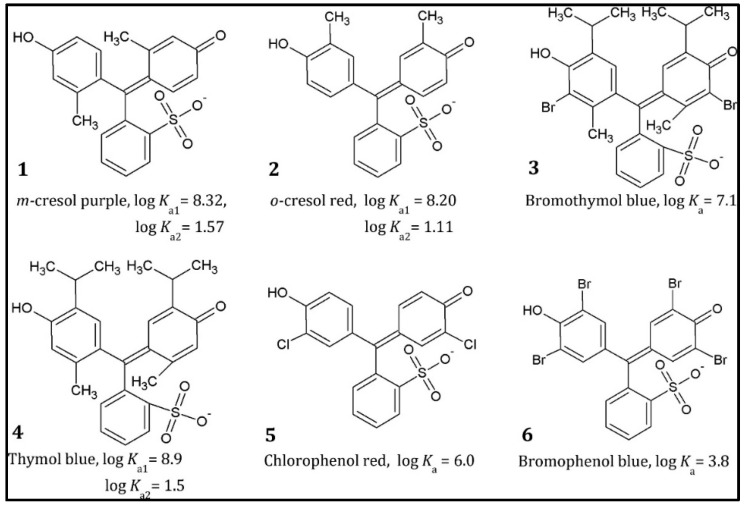
Dyes were used as sensing moieties. Reprinted with permission from the reference [73]. Copyright 2021 American Chemical Society.

The basis of this sensing approach is the quenching of luminescence by Cu^2+^. Because nitrogen likeliest correlates with the metal cation, an electron shift from pyrene to Cu^2+^ occurs, encouraging nonradiative relaxation. In the presence of Fe^3+^ and Hg^2+^ in the suspension stage, measurements were purchased. These cations produced a modest level of gleaming quenching. As a result, the suggested approach may be regarded as selective against Cu^2+^ in solution [48]. Magnaghi et al. [73,74] created an intriguing novel polymeric optode for high-protein food deterioration based on an EVOH copolymer. In this work, EVOH was functionalized with several dyes (Figure 8), which caused the color to change at different pH levels.

The sensors alter color based on the ambient pH in which they are placed. Likewise, Alberti et al. [75,76] generated a sensor for Fe(III) in which EVOH was utilized as a solid state. Deferoxamine meylate (DFO) and 3,4-hydroxypyridinone ligand (KC18) functionalized the copolymer. These ligands tend to form tinted composites with Fe(III).

**Figure 9:**
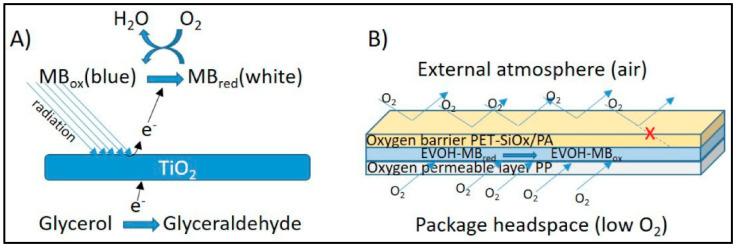

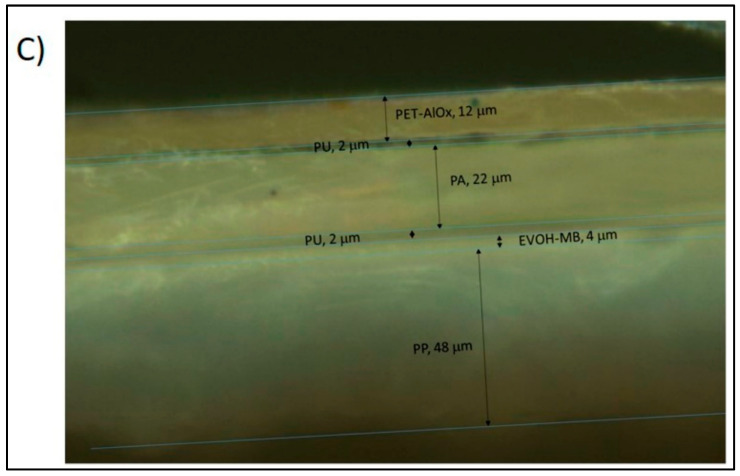
Schematic mechanism of O_2_ detection. (**A**) Scheme of the functioning process (**B**) multilayer assembly of the sensor, and (**C**) Image of the multilayer structure of the oxygen sensor. Reprinted with permission from the reference [77]. Copyright 2019 MDPI.

Lopez-Carballo et al. [77] created an O_2_ chromatic sensor using blue methylene, glycerol, TiO_2_, and EVOH. To protect the content, the presence of oxygen within food packaging must be avoided. The researchers combined TiO_2_, glycerol, and methylene blue in the EVOH network to create a detecting solid phase. The resulting substance was utilized to create layers and coatings. The sensing procedure is depicted in Figure 9.

EVOH can be effectively used in the development of chemical sensors, especially as a brace for acquiring extrudable substances, which, in theory, is appropriate for experimental utilization because of its tendency to liquefy in hydro-alcoholic blends and applied to pliable substrates via coating or printing processes.

### 3.5. Polymer Inclusion Membranes

Polymer inclusion membranes (PIMs) are liquid membranes based on polymers, invented over 50 years ago, and are employed as the recognizing component of ion-selective electrodes and ocular detectors (optodes). PIMs have recently been used in specimen development and preconcentration, passive sampling, and may be integrated into networked and self-operating devices. A PIM is made of a liquid state and a polymeric brace, which is commonly PVC, cellulose triacetate, or polyethylene glycol (vinylidene fluoride-co-hexafluoropropylene). The polymeric component serves as the membrane’s skeleton, providing mechanical strength. An extractant (carrier) in the liquid phase holds the analyte via ion–couple development or complex formation. Some conveyors have plasticizing properties; however, during membrane production, an extra conditioner is introduced to improve elasticity or increase the solubility of the analyte in the liquid state. PIMs are generally made by liquefying all the ingredients in a tiny amount of evaporative solvent (dichloromethane or tetrahydrofuran) and diffusing them. The solvent was gently evaporated until a homogenous and clear PIM on the fling surface, which might be level or columnar, was produced. An ion-choosy electrode can be created by casting a film on the tip of the electrode. By including a chromophore in the membrane composition, flat-sheet PIMs may be employed to create optical sensors [78,79]. The gold nanomaterial polymer inclusion membrane was used as a recognizing stage, as well as to immobilize the immunizer. The suggested sensor has the capability for in-place food checks due to its easy investigational technique and the mobility of potentiometric apparatus. The proposed platform’s strong performance was proven by an operating span of 1.3–13 × 10^6^ cells mL^−1^ and an LOD of 6 cells mL^−1^, which were equivalent to previous electrochemical label-free immunosensors for ST. The suggested technique may be used for any bacteria–antibody coupling by simply altering the particular antibody and adjusting the AuNP-PIM. For Zn(II) determination, a disposable optode was designed [45]. It is made by immobilizing a dye, 2-acetylpyridine benzoyl hydrazone (2-APBH), in a polymer insertion sheet adhered to the outer side of a polyester band. An investigational factorial blueprint is used to optimize the sheet constitution to get a substance with a pleasing look, as well as appropriate concrete and visual qualities. The best sheet was made of 2.5 g PVC, 4 mL tributyl phosphate, and 40 mg 2-APBH. The optical sensor has a level span of 0.03 (i.e., the LOD) to 1 mg L1 of Zn(II), and it reacts for almost 30 min when submerged in aqueous solutions with a pH of 6. The reactivity to Zn(II) is superior to that of other ordinary cations. The instrument is used to determine Zn(II) levels in an authenticate reference material, perforated tap water, mineral drinks, food amalgamators, and foot health protection items, yielding consistent findings. This section emphasizes the rising interest in PIMs in chemical sensing. The fabrication of tiny PIM-rooted tools with inflated reactivity and suitability for environmental, biological, and clinical study will be a future challenge [80].

### 3.6. Polymer Composites and Nanocomposites

Polymer nanocomposites (PNCs) are formed by the fusion of a polymeric phase (continuous) with a discontinuous phase of nanofillers. Numerous advantages in mechanical, optical, and electrical qualities have piqued the interest of experts all around the world. PNCs may be manufactured in a variety of forms, making them ideal for the development of chemical and biological sensors. Nanostructured polymers have a significant influence on biological and technological fields, particularly in drug delivery, catalysis, and sensing applications [81,82]. Table 1 shows the PNCs are good candidates for electrochemical sensor development due to their high electrical conductivity, huge surface area, and quick electron rate. They comprise inorganic nanomaterials in combination with conducting polymers/CNTs/graphene [83,84].

**Figure 10:**
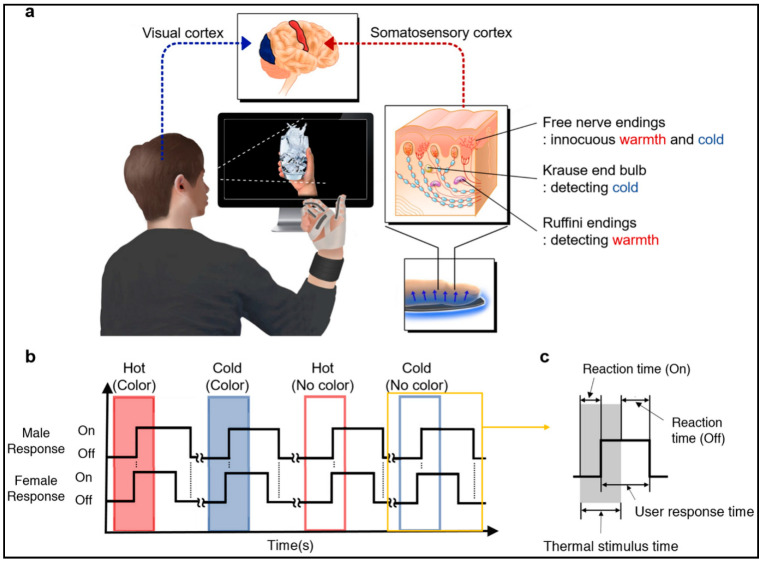
Thermal display glove for interacting with virtual reality. (**a**) Illustration of the operation of thermal display glove system, (**b**) Response time to the thermal and color stimuli in the user test. (**c**) Definition of reaction time to the thermal stimulus. Reprinted with permission from reference [86]. Copyright 2020 Nature.

Recent breakthroughs in nanotechnology/wearable devices have emerged as significant progress in health care and medical diagnostics, robotic systems, prostheses, visual realities, and professional sports. The use of wearable sensors for the detection of motion and body signals is done by attachment to clothing or fastening to human skin with adhesive straps [85]. An intriguing paper [86] revealed that piezoresistive sensors can be developed as strain sensors employing a copolymer of (styrene butadiene styrene) as the matrix, supplemented with conducting media in the form of carbon nanofillers. Extrusion or spray printing processes were used to create these sensors, which enabled scaling up and inclusion in novel devices. Figure 10 depicts a thermal display glove system, which has a linkage between a physiological process and a virtual condition.

Graphene is a newly discovered two-dimensional carbon substance. Its clever electrical, optical, and mechanical qualities make it a good candidate for strain sensors. Chun et al. [87,88] developed graphene-based sensors on a stretchy polydimethylsiloxane substrate that can detect microscopic stresses (up to 0.1%) and provide unique output signals. This sensor can detect stretching, bending, and torsion in the human body. The efficient coupling of diverse nanomaterials and conducting polymers with conductive polymers offers new avenues for using PNCs in high-performance biosensing and electrochemical sensing [89]. 

**Table 1:** Table summarizing various polymers/polymer nanocomposites, analytes, immobilization techniques, and LOD.

S. No.	Polymer/Nanocomposite	Analytes	Techniques of Immobilization	Detection Limit	References
1.	Polyaniline	Urea/Glucose	Encapsulation	-	[90]
2.	Polypyrolle	Glucose	Cell coating	0.005 mM	[91]
3.	Polyaniline	*E. coli*	Covalent linkages	10 CFU/mL	[92]
4.	Polypyrolle	Catechol	Entrapment	1.8 µM	[93]
5.	Ferrocene Polypyrolle	*M. tuberculosis*	-	1 aM–100 fM	[94]
6.	Polyaniline/MWCNT-Polymethylene blue	Cardiac troponin T	-	0.10 to 8.0 pg/mL	[95]

### 3.7. Sensors for Antioxidant Activity

The expected substances for the formation of electrochemical sensors are natural electropolymerized phenolic antioxidants. Electropolymerization of the antioxidants of these substances enables the development of heat-proof polymers. Electro-agile polymeric films capable of functioning as redox-arbitrators and furnishing an electrocatalytic action on the oxidation of organic substances are formed of phenolic antioxidants comprising catechol specks in their composition. To get the extraordinary feedback of the intent analyte, the electropolymerization circumstances need to be optimized. The conduction of innate phenolic antioxidant substances can be accelerated by integrating them with conveying substances such as carbon nano substances, metals, or metal oxide nanomaterials. This perspective has a positive influence on electroconductivity, as well as the efficient outer area of the detector on which polymer electroplating is done. The integration of reformed films elevates the reactivity and specificity of the intent analyte feedback. Moreover, display-imprinted electrodes with already accumulated nano substances of carbon, metal, or oxides of metals can be utilized for restricting polymeric coverage, which is beneficial for day-to-day investigation. This ultimately leads to a decrease in sensor synthesis time, and the analysis procedure. The poriferous shape of polymers offers different advantages, enabling their use as a reactive film of the detector that provides feedback to a broad spectrum of biological and inanimate substances. The polymeric sheets can work as a semipermeable layer, thus allowing the formation of electrochemical detectors for substances of less molecular weight, such as hydrogen peroxide, nitric oxide, and ascorbic acid. The existence of electron-giving atoms in the polymeric layered construction enables it to adhere to heavy metals, furnishing their preconcentration on the outer side of the detector. The perforated setup of the polymeric coverage of organic analytes has the potential to adsorb specimens due to the constructional uniformity and size outcome, specifying both the single or batch reactivity of the detector produced and their broad implementation area in electroanalysis. The establishment of innate phenolic antioxidants and all antioxidant criteria is of peculiar interest and has a notable experimental perspective.

## 4. Use of Polymers in Biosensing and the Role of Cellulose in Biomarker Detection

Biomarkers are considered biologically existing molecules, a gene by which a particular disease or pathological condition can be detected. Cancer biomarkers are studied as a subcategory and can be defined as a biofluid or any biological blood entity that distinguishes between a normal and abnormal biological state [96]. Cellulose paper, owing to its promising thermal and mechanical strength and highly biocompatible nature, makes it a promising candidate for the designation of a non-invasive diagnosis of several disease biomarkers. In the case of polymer-based biosensors (PBBs), cellulose paper is utilized as semi-rigid/rigid scaffold possessing numerous pores enabling storage of immobilized reagents [97]. Das et al. [98] reported work on modified cellulose for cancer biomarker detection using fluorescent spectroscopy. The modified cellulose-based assay detected epithelial cell adhesion molecules both in the buffer and about 10% of bovine solution, utilizing reaction times of less than half an hour. In another study, Lin et al. [99] reported cellulose paper functionalized by dopamine for use as a visualized biosensor. The developed biosensor exhibited some indispensable properties, such as portability, disposability, and visible characteristics, in comparison to other known biosensors. Some paper-based sensors have also been used as immunosensors in pregnancy testing and the evaluation of blood glucose levels. The prime challenge for this type of sensor is scant concentration of cancer biomarkers at early stages of the tumor, and efforts are being made to achieve higher sensitivity by tuning the signal amplifications. The paper-based sensing devices being able to handle easily pose several challenges. The serious challenge is maintaining a higher bioactivity of the recognition molecules of cellulose paper-based devices. As a result, antibodies, and enzymes become prone to oxidation when moist, which are otherwise protected from oxidation under dry conditions.

## 5. Grand Challenges

Polymer-based sensors have been found to face the following challenges:

i. In search for selection of right precursors, followed by refining and devising the mechanisms of reaction and mechanisms that allow a measurable signal detector to be obtained;
ii. Searching novel materials for incorporation as polymer matrices;
iii. Development of pH based and ion-selective sensors;
iv. Doing trails for automating the process of procurement of polymer-based sensors.

However, research on sensors based on polymers is ongoing, and new solutions are sought to fully understand the mechanism of the reaction. Majumdar and Adhikari discussed various trends that could prove to be of prime importance when evaluating the work done for polymer-based sensors in the upcoming years:

i. Utilization of chemical alterations for immobilization improvement of receptor molecules.
ii. Search of novel techniques and smart materials for their processing.
iii. Development of sensing materials capable of responding to various stimuli in distinct ways.
iv. Investigating the biochemical reactions in non-aqueous media.

The use of polymers in sensing applications is not restricted to softness, lightweightness, flexibility, low production costs, and longer shelf lives. Moreover, by changing the nanomaterial dimensions, the property of sensing can be increased up to a considerable limit to attain a calculated limit of detection and high sensitivity.

## 6. Concluding Remarks

Taking all the parameters into consideration, polymer-based sensors incorporating appropriate modifications or specific functionalities may be used as highly specific sensors. The use of polymers in sensing can be either directly or by immobilization of specific receptors onto them. The benefits of using polymers as sensors are not restricted to their ease of functionalization, biocompatibility, longer lifetimes, lightweightness, softness, and cost-effective production. There has been a focus on significant modifications for better performance and to take the sensors from the lab into the device marketing applications. Collaboration at interdisciplinary levels between computational scientists, engineers, and chemists will provide a boost in the implementation of large-scale production and development to overcome these obstacles.

## 7. Future Perspectives

Although much work has been reported in the field of sensing materials, there is still some unexplored area in which work needs to be done. The need to develop sensors possessing a good limit of detection (LOD) and higher order of selectivity/sensitivity has a specific need in pharmaceutics, herbicides, and pesticides. Moreover, conducting polymer-based/MO nanofiller-based nanocomposites are still unexplored. The nanocomposites based on transition metals/metal oxides can serve the purpose of biosensor/electrochemical sensor both as a result of their prominent color change nature.

## Data Availability

Not applicable.
